# Polymerization Behavior of New Stela Self-Cured Bulk-Fill and Light-Cured Composites: An In Vitro Comparative Study

**DOI:** 10.3390/jfb17100509

**Published:** 2026-10-10

**Authors:** Rasha Haridy, Joud Alshahrani, Maha Alfarraj, Bashayer Alfahed, Dana Alsenan, Abrar Bin Nooh, Sarah Alghamdi, Abdullbasit Bugshan, Amir Msmar

**Affiliations:** 1 Department of Clinical Dental Sciences, College of Dentistry, Princess Nourah bint Abdulrahman University, P.O. Box 84428, Riyadh 11671, Saudi Arabia; saaalghamdi@pnu.edu.sa; 2 Internship Unit, College of Dentistry, Princess Nourah bint Abdulrahman University, P.O. Box 84428, Riyadh 11671, Saudi Arabia; joudsaad44100@gmail.com (J.A.); alfarrajmahaa@gmail.com (M.A.); 3 Dental Clinics Department, King Abdullah bin Abdulaziz University Hospital, Princess Nourah bint Abdulrahman University, P.O. Box 84428, Riyadh 11671, Saudi Arabia; 4 Department of Dental and Oral Rehabilitation, College of Dentistry, King Saud University, P.O. Box 60169, Riyadh 11545, Saudi Arabia; aaalodayni@ksu.edu.com; 5 Statistics Unit, Deanship of Scientific Research, Imam Abdulrahman Bin Faisal University, P.O. Box 1982, Dammam 31441, Saudi Arabia; ahmsmar@iau.edu.sa

**Keywords:** degree of conversion, bulk-fill composite, chemical cure composite, nano-hybrid composite, Fourier-transform infrared spectroscopy, polymerization kinetics

## Abstract

Adequate polymerization is essential for the mechanical performance and longevity of resin-based composites; however, recently introduced chemical cure, bulk-fill composites remain underexplored. This in vitro study compared the degree of conversion (DC) of a chemical cure bulk-fill composite (Stela, SDI) with those of a nano-hybrid light-cured composite (Filtek™ Z250, 3M). Sixty standardized disc-shaped specimens (5 mm × 2 mm; *n* = 30 per material) were prepared according to the manufacturer’s instructions. We measured DC using Fourier-transform infrared spectroscopy (FTIR) immediately after polymerization and at 5, 10, 20 min, and 24 h. Both materials showed a progressive increase in DC over time. However, the light-cured composite consistently achieved higher conversion values, reaching 62.39 ± 4.23% after 24 h compared with 47.58 ± 3.04% for the chemical cure composite (*p* < 0.001). These findings indicate that DC alone should not be considered predictors of composite performance.

## 1. Introduction

Resin-based composite materials have become a cornerstone of contemporary restorative dentistry because of their excellent aesthetics, favorable clinical performance, and support for conservative tooth preparation [1,2,3]. Growing clinical reliance on these materials is driven by ongoing advances in resin chemistry and filler technology that aim to improve mechanical performance and long-term durability [4,5]. However, the clinical success of composite restorations still depends heavily on adequate polymerization, as insufficient curing adversely affects mechanical properties and increases the risk of marginal degradation and restoration failure [6,7].

Polymerization converts carbon-carbon double bonds (C=C) in resin monomers into a polymer network, commonly quantified as the degree of conversion (DC), which is a key determinant of material performance [8,9]. Higher DC is associated with improved mechanical strength, enhanced wear resistance, and reduced release of unreacted monomers that may be toxic [10,11,12,13].

Nano-hybrid resin composites mark a significant advance in restorative materials by combining nano-sized fillers with conventional particles, thereby improving mechanical properties, polishability, and aesthetics [14,15]. These materials are predominantly light-cured and depend on photoinitiator systems that require sufficient light irradiance and penetration to achieve adequate polymerization throughout the restoration [16,17]. However, light attenuation remains a major limitation of light-cured systems, as increased restoration thickness, darker shades, and higher filler content can reduce light transmission and compromise curing efficiency in deeper layers [18,19,20].

To address these limitations, bulk-fill composites were introduced, enabling placement in thicker increments while maintaining acceptable polymerization and mechanical performance [21,22]. Systematic reviews indicate that bulk-fill composites can achieve clinical performance comparable to conventional composites when used within recommended clinical parameters [23,24]. Despite these improvements, light-cured bulk-fill materials may still show depth-dependent variations in polymerization, particularly in deeper regions where light irradiance is reduced [25,26].

Chemical cure composites have been proposed as an alternative to overcome light-attenuation limitations because polymerization is initiated by a chemical reaction rather than by light activation [27]. This mechanism theoretically enables more uniform polymerization regardless of restoration depth or optical properties [28]. Stela (SDI) is a recently introduced chemical cure bulk-fill composite designed to provide consistent polymerization without relying on external light sources, addressing a major limitation of light-cure systems [29,30,31].

Unlike photoactivated materials, chemical cure composites exhibit distinct polymerization kinetics, typically marked by a slower, continuous conversion that can affect both immediate and long-term material properties [32,33]. In contrast, light-cured nano-hybrid composites polymerize rapidly upon light exposure, yielding high early mechanical strength but potentially limited penetration in deeper regions [34,35]. Inadequate polymerization has been linked to reduced surface microhardness, increased wear, marginal breakdown, and greater susceptibility to secondary caries [36,37].

Therefore, evaluating DC over time is essential for understanding the polymerization behavior and mechanical performance of composite materials [38,39]. Although nano-hybrid light-cured composites have been extensively investigated, evidence on the polymerization behavior of recently introduced chemical cure bulk-fill composites remains limited [40]. We hypothesized that DC would differ between the two investigated materials over the designated polymerization time points. Therefore, this study aimed to compare the DC of the two materials over designated polymerization time points.

## 2. Materials and Methods

### 2.1. Study Design and Sample-Size Calculation

This in vitro study compared the polymerization behavior of two resin-based composites with different curing mechanisms: a chemical cure bulk-fill composite and a light-cured resin composite. The primary outcomes were DC; a total of 60 standardized disc-shaped specimens were fabricated and divided into two groups based on the restorative material, with 30 specimens in each group.

We evaluated the adequacy of the final sample size through a sensitivity analysis IBM SPSS (version 27.0, IBM Corp., Armonk, NY, USA). With 30 specimens per group, a two-sided significance level of 0.05, and 80% power, the study could detect a standardized between-group effect of Cohen’s d = 0.74.

### 2.2. Materials

The study evaluated two commercially available resin-based composites with different polymerization mechanisms. A chemical cure bulk-fill (Stela, SDI Limited, Bayswater, Victoria, Australia) and a nano-hybrid light-cured composite (Filtek™ Z250, 3M ESPE, St. Paul, MN, USA) were selected to compare their polymerization behavior under standardized laboratory conditions. Specimen preparation and testing were performed strictly in accordance with the manufacturers’ instructions. Table 1 summarizes the characteristics of the materials. Both materials were stored under the manufacturers’ recommended conditions before specimen preparation and were used before their expiration dates.

### 2.3. Specimen Preparation and Group Allocation

**Figure 1:**
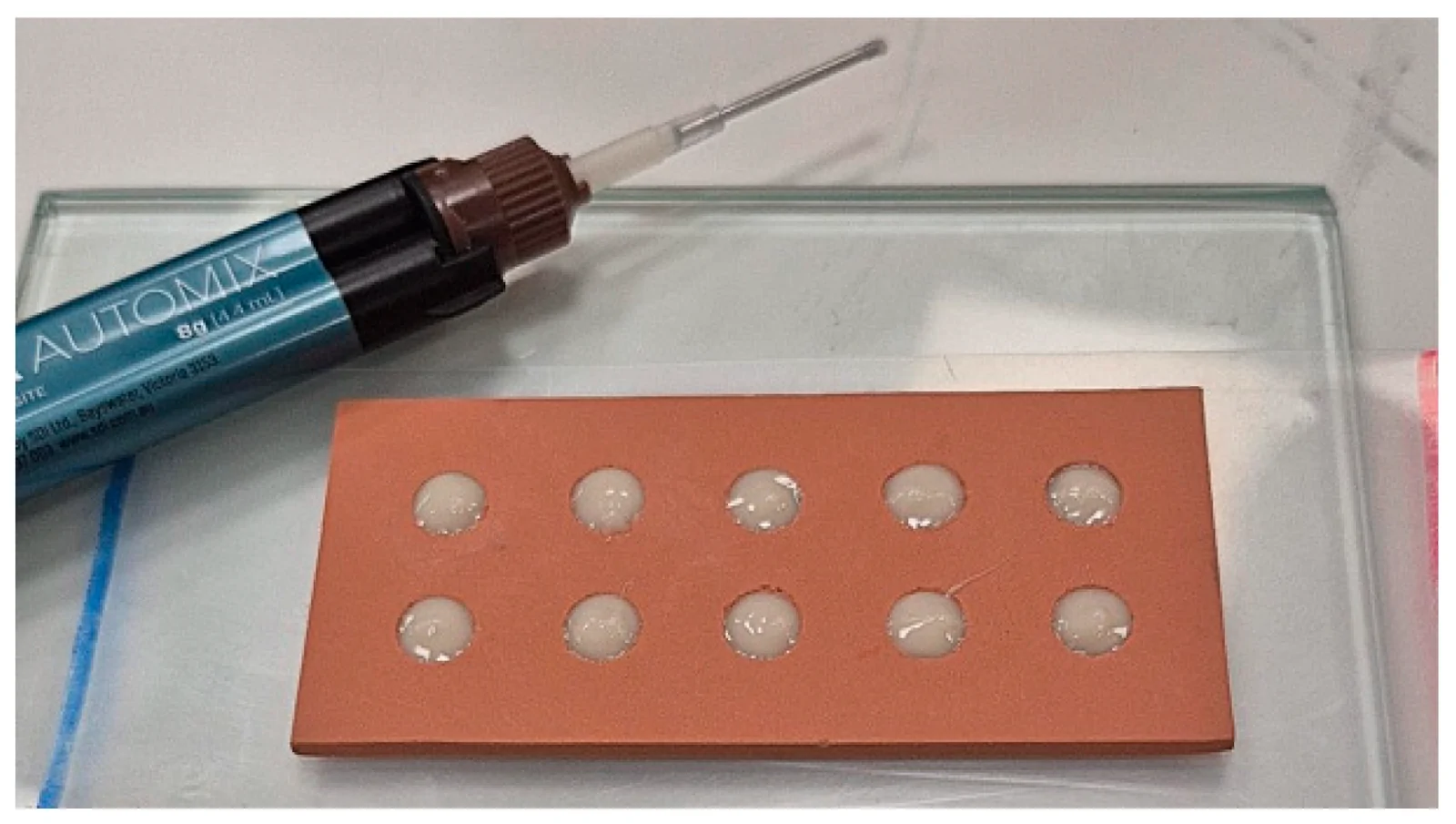
Chemical cure composite specimen preparation.

**Figure 2:**
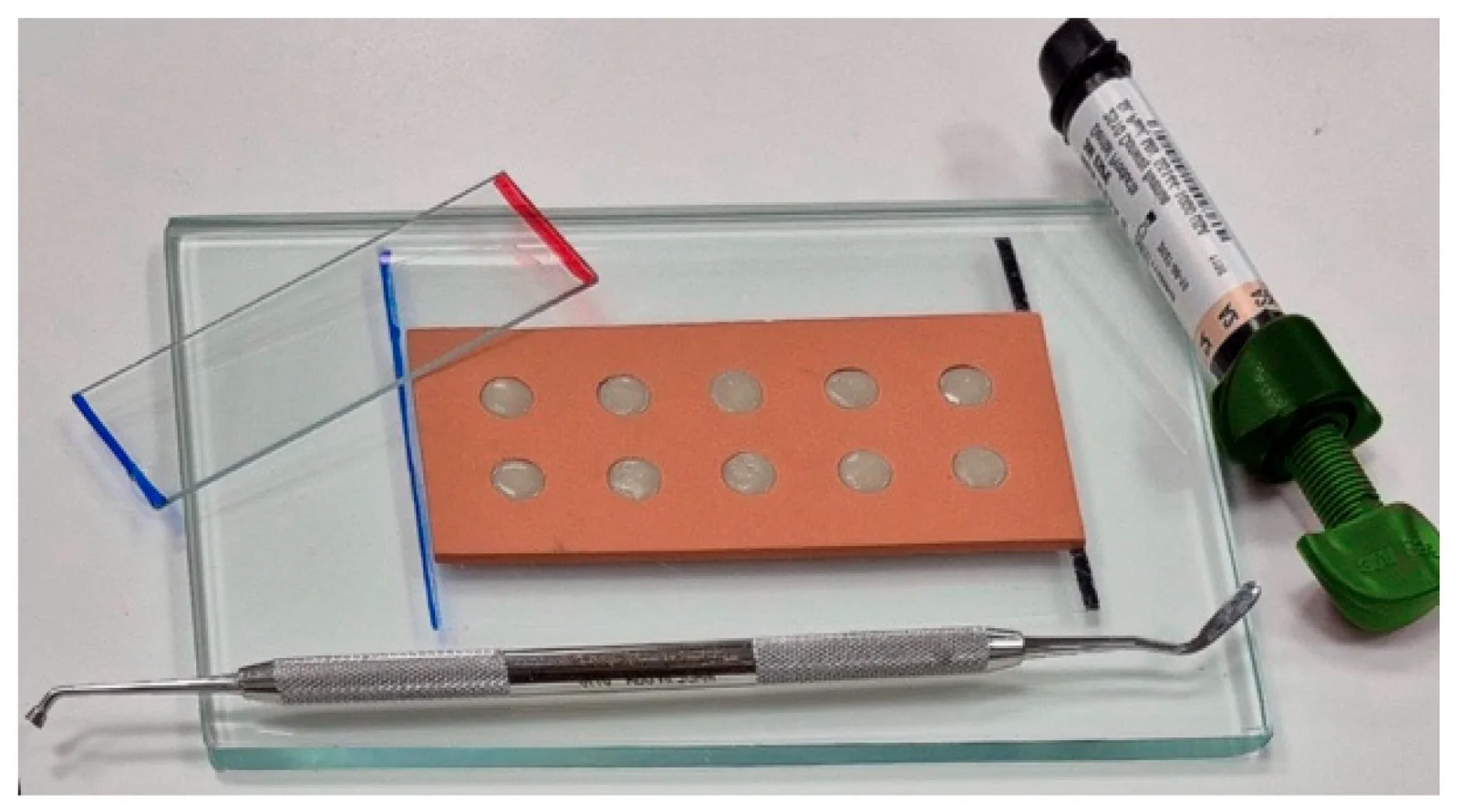
Light-cure composite specimen preparation.

A total of 60 standardized disc-shaped specimens were fabricated using a cylindrical polytetrafluoroethylene (PTFE) mold measuring 5 mm in diameter and 2 mm in thickness. Specimens were equally allocated to two experimental groups (*n* = 30 per group) based on the restorative material evaluated: Group CC (Stela; SDI) and Group LC (Filtek™ Z250; 3M). The PTFE mold was positioned on top of the FTIR reading surface and material was dispensed directly on the crystal. A glass slab was used on top of the specimen mold, which was covered with a transparent polyester strip. The composite material was placed into the mold as a single increment with care to minimize air entrapment. Gentle manual pressure was applied to extrude excess material and obtain a flat, uniform specimen surface, as shown in Figure 1 and Figure 2.

After polymerization according to the respective curing protocol, each specimen was carefully removed from the mold and visually inspected under magnification with loupes for defects, including voids, fractures, and surface irregularities. Defective specimens were discarded and replaced. The same operator prepared all specimens to minimize operator-dependent variability. Each specimen was assigned an identification code before testing to ensure traceability throughout the experimental procedures.

### 2.4. Polymerization Procedures

#### 2.4.1. Chemical Cure Composites

Stela (SDI) was dispensed using the manufacturer’s automixing system and immediately inserted into the mold. After placement, the material was allowed to polymerize undisturbed for 5 min in accordance with the manufacturer’s instructions. During polymerization, the specimen remained covered with a polyester strip and a glass slide to maintain standardized specimen thickness and obtain a flat surface.

#### 2.4.2. Light-Cured Composites

**Figure 3:**
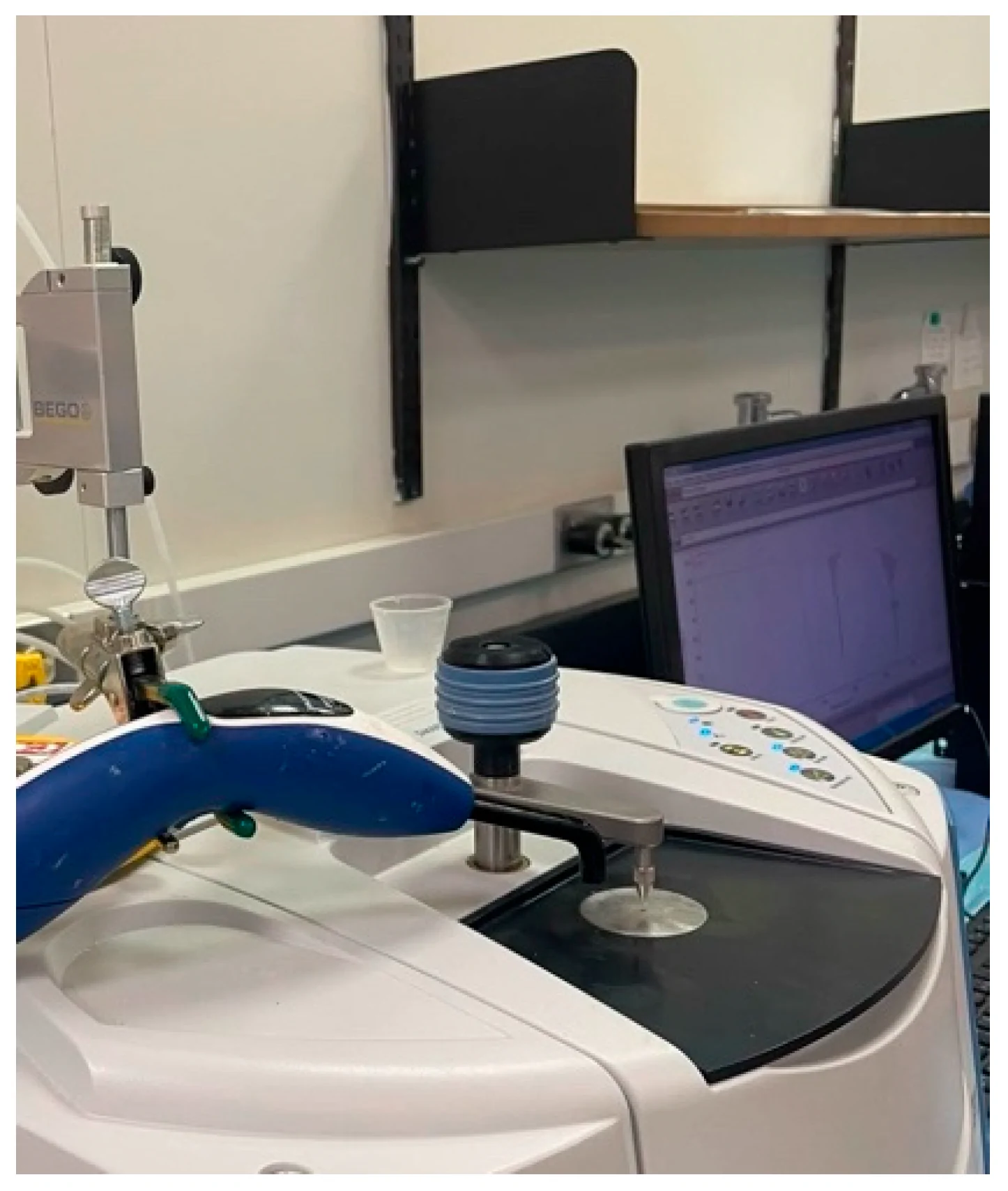
Curing unit set; standardized curing distance.

Filtek™ Z250 (3M ESPE, St. Paul, MN, USA) was photoactivated with an LED light-curing unit (Bluephase Meter II, Ivoclar Vivadent AG, Schaan, Liechtenstein) for the manufacturer’s recommended exposure time (40 s). The curing unit delivered an irradiance of 1500 mW/cm^2^, which was verified using a Bluephase Meter radiometer (Ivoclar Vivadent AG, Schaan, Liechtenstein) before specimen preparation. The light-guide tip was positioned perpendicular to the specimen throughout light activation to minimize curing distance, as shown in Figure 3. After polymerization, the specimens were removed from the mold and allowed to cool to room temperature before further processing.

### 2.5. Degree of Conversion (DC) Assessment

The DC was determined using attenuated total reflectance Fourier-transform infrared spectroscopy (ATR-FTIR) on a VERTEX 70 FTIR spectrometer (Bruker Optics, Ettlingen, Germany). Spectra were collected from the top surface of each specimen over a spectral range of 1450–1650 cm^−1^ at a resolution of 4 cm^−1,^ with 64 scans per measurement. A background spectrum was obtained before each session in accordance with the manufacturer’s recommendations.

For each material, the unpolymerized reference spectrum (T0) was obtained from a separate aliquot of material rather than from the experimental specimens. For Filtek Z250, the reference spectrum was obtained before light activation. For Stela, because chemical polymerization is initiated upon automixing, a strictly pre-polymerization spectrum could not be obtained after mixing. Therefore, a separate freshly mixed aliquot was placed on the ATR crystal and the reference spectrum was acquired immediately after dispensing, with spectral acquisition initiated without delay to minimize conversion before measurement. This spectrum was used solely as the unpolymerized reference for calculations and was not considered an experimental post-initiation time point or included in the statistical analysis. T0 therefore represented the material-specific unpolymerized reference condition rather than a post-initiation DC assessment time point.

For Filtek Z250, the material-specific immediate spectral acquisition was initiated immediately after completion of the 40 s light-curing cycle. For Stela, spectral acquisition for the material-specific immediate measurement was initiated after automixing and dispensing immediately, with the freshly mixed material placed directly on the ATR crystal. Spectral acquisition was initiated approximately after 10 s of dispensing. Because chemical polymerization begins upon mixing, this measurement represented an early chemically initiated polymerization stage rather than a stage directly equivalent to the post-light-activation measurement of Filtek Z250. Accordingly, the material-specific immediate measurements were not considered directly comparable between the two materials and were not included in the between-material repeated-measures statistical analysis. The first shared time point used for direct between-material comparison was therefore 5 min.

The DC was calculated using the following equation:

DC (%) = [1 − (A1638/A_ref_) polymerized ÷ (A1638/A_ref_) Unpolymerized] × 100

In this equation, A1638 represents the absorbance of the polymerization-sensitive aliphatic C=C absorption band at approximately 1638 cm^−1^ whereas A_ref_. represents the material-specific internal reference band. For Filtek Z250, which contains the aromatic monomers Bis-GMA and Bis-EMA, the aromatic C=C band at approximately 1608 cm^−1^ was used as the internal reference. For Stela, whose resin matrix is UDMA-based and lacks the corresponding aromatic resin structure, the amide II band at approximately 1537 cm^−1^ was used as the internal reference. The amide II band has been validated as a stable internal reference for FTIR determination of monomer conversion in UDMA-based dental resin [41,42]. The A1638/A_ref_ polymerized ratio was obtained from the specific T0 reference aliquot, whereas the A1638/A_ref_ polymerized ratio was obtained from the corresponding experimental specimen at each post-initiation time.

### 2.6. Statistical Analysis

Statistical analyses were conducted using IBM SPSS (version 27.0, IBM Corp., Armonk, NY, USA). Each specimen served as the experimental unit. We summarized continuous data as means and standard deviations, with 95% confidence intervals reported where appropriate. DC data were analyzed with a two-way mixed-design repeated-measures analysis of variance, with material type (chemical cure and light-cured composites) as the between-subject factor and polymerization time as the within-subject factor. The repeated-measures analysis included the four shared assessment times of 5 min, 10 min, 20 min and 24 h. T0 was excluded because it represents the unpolymerized reference spectrum used for DC calculations rather than a post-initiation outcome. The material-specific immediate measurements from the direct between-material analysis were also excluded because they represented different stages of polymerization and were therefore not considered directly comparable. No separate immediate DC value for Stela was included in the statistical analysis; the first standardized post-initiation DC assessment for Stela included in the comparative analysis was 5 min. The main effects of material and time, as well as the material-by-time interaction, were evaluated. Significant effects were further examined using pairwise comparisons.

## 3. Results

**Figure 4:**
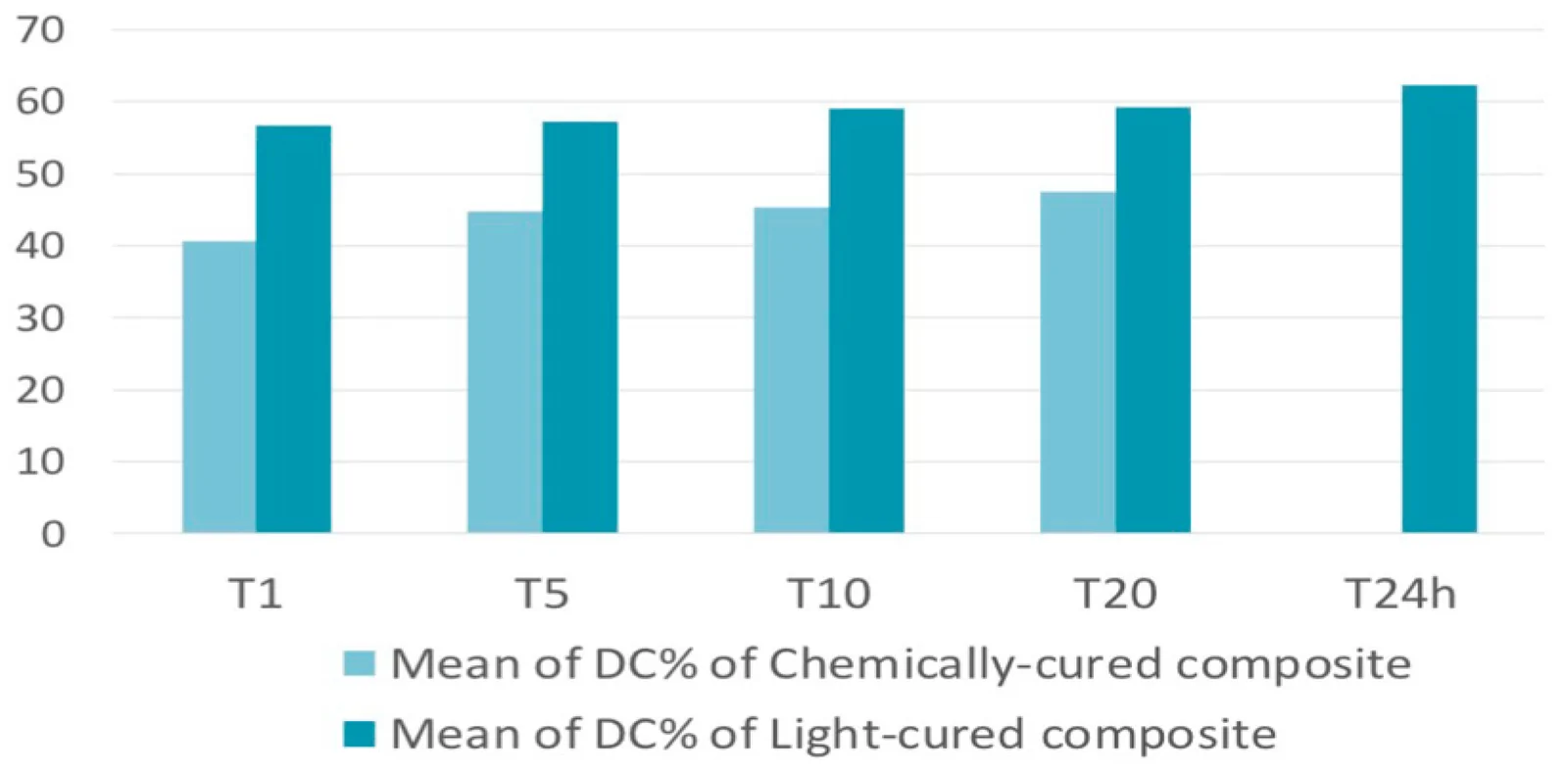
Mean DC time of different materials.

The mean DC increased over time in both materials (Table 2 and Figure 4). In the light-cured composite, DC increased from 56.78 ± 4.62% at the material-specific immediate assessment following photoactivation to 62.39 ± 4.23% at 24 h. For Stela, the material-specific immediate spectrum was acquired approximately 10 s after dispensing; however, this measurement represented an early post-mixing condition that was not equivalent to the immediate post-irradiation measurement of Filtek Z250. The measurements were not compared statistically because they represented distinct stages of polymerization. Direct between-material comparison, therefore, began at the first shared assessment time of 5 min. At all shared assessment times, the light-cured composite demonstrated higher mean DC values than the chemical cure composite.

Between-material comparisons showed that the light-cured composite had significantly higher DC than the chemical cure composite at every shared assessment time. The estimated mean differences were 16.78 percentage points at 5 min (95% CI, 14.93–18.64; *p* < 0.001), 14.27 percentage points at 10 min (95% CI, 12.28–16.26; *p* < 0.001), 13.97 percentage points at 20 min (95% CI, 11.96–15.98; *p* < 0.001), and 14.81 percentage points at 24 h (95% CI, 12.90–16.71; *p* < 0.001).

For ratio1 analysis, the light-cured composite increased gradually from 56.78 ± 4.62% at its material-specific immediate assessment following completion of light activation to 62.39 ± 4.23% after 24 h. In contrast, the chemical-cured composite increased from 40.57 ± 2.22% at 5 min to 47.58 ± 3.04% at 24 h. Because the material-specific immediate measurements represented different stages of polymerization, they were not directly compared. T0 represents the material-specific unpolymerized reference.

The light-cured composite consistently achieved higher DC values at all corresponding time points. The mean differences between materials were 16.78% at 5 min, 14.27% at 10 min, 13.97% at 20 min, and 14.81% at 24 h, with all comparisons reaching statistical significance (*p* < 0.001).

The light-cured composite showed rapid early conversion immediately after photoactivation, followed by a more gradual increase during the post-cure period. Conversely, the chemical cure composite exhibited a slower but continuous increase in conversion over time, reflecting the progressive nature of redox-initiated polymerization reactions. Although the conversion gap between the materials decreased slightly over time, the chemical cure composite did not reach the conversion levels achieved by the light-cured material within the 24-h observation period. This finding suggests that polymerization differences persisted despite ongoing maturation of both materials. The overall increase in DC from the earliest to the latest measurement points confirms continued post-polymerization in both restorative systems. These findings highlight the dynamic nature of composite polymerization and emphasize that final material properties may continue to develop beyond the first curing stage.

## 4. Discussion

The present study compared the polymerization behavior of a chemical cure bulk-fill resin composite, Stela, and a conventional light-cured nano-hybrid resin composite, Filtek Z250. The principal findings were that the light-cured composite demonstrated significantly higher DC values at all shared assessment times compared to the chemical cure composite. Both materials showed continued conversion over time, although the difference remained evident at 24 h. Therefore, the study accepted the hypothesis that polymerization behavior differs by material.

The DC of Filtek Z250 increased from 56.78 ± 4.62% immediately after photoactivation to 62.39 ± 4.23% at 24 h. This pattern aligns with the established polymerization behavior of light-cured dimethacrylate-based composites in which substantial monomer conversion occurs during light exposure, followed by additional post-cure conversion over time [43,44,45]. Continued post-cure conversion has been attributed to the persistence and mobility of reactive free radicals and unreacted monomer within the polymer network, allowing free-radical propagation to continue after light exposure has ceased [46,47]. Several factors influence the magnitude and rate of conversion in photoactivated composites, including the photoinitiator system, resin-matrix composition, radiant exposure, light spectrum, specimen thickness, and filler-related light scattering [48,49]. In the present study, the standardized 2 mm specimen thickness and the use of a high-irradiance LED curing unit likely facilitated photoactivation of Filtek Z250 at the measured top surface.

The DC of Stela increased from 40.57 ± 2.22% at 5 min to 47.58 ± 3.04% at 24 h, indicating that polymerization continued beyond the manufacturer-specified setting period. Chemically activated resin composites rely on chemical initiation rather than external light exposure and may therefore exhibit slower conversion kinetics than photoactivated materials [50,51]. The continued increase observed for Stela is consistent with progressive radical formation and post-setting network development. Nonetheless, its DC remained significantly lower than that of Filtek Z250 at all shared assessment times, with a mean difference of 14.81 percentage points at 24 h. This finding indicates that the absence of light dependence does not necessarily result in a higher surface DC during the first 24 h.

The DC recorded for Stela should not be attributed solely to its chemically activated curing mechanism. The investigated composites differed in resin composition, filler type, filler loading, initiator system, and material classification. Filtek Z250 contains Bis-GMA, UDMA, and Bis-EMA and has a reported filler loading of 82 wt%. In contrast, Stela is based primarily on UDMA and glycerol dimethacrylate and has a lower reported filler loading of 61.2 wt% [52,53]. Monomer viscosity, molecular flexibility, functional group concentration, and interactions between the organic and inorganic phases can influence monomer mobility and the final extent of conversion [54]. Consequently, the observed between-material difference reflects the combined influence of curing mechanism and material composition rather than an isolated effect of chemical versus light activation. Two resin composites may therefore demonstrate relatively similar conversion values while exhibiting substantially different mechanical properties because their polymer networks and inorganic phases differ.

The present findings support the combined use of chemical and mechanical assessments when characterizing polymerization performance. The findings should also be interpreted in the context of the measurement location. ATR-FTIR spectra were obtained from the standardized bottom surface of 2-mm specimens. The results therefore characterize surface polymerization under controlled laboratory conditions and should not be taken as direct evidence of uniform polymerization throughout clinically placed bulk increments.

Although chemically activated systems do not depend on light transmission, assessment at different specimen depths remains necessary to confirm whether conversion is spatially uniform. From a clinical perspective, the higher DC of Filtek Z250 suggests favorable surface polymerization. However, these findings should not be interpreted as evidence of overall clinical superiority. Stela is intended to provide chemically initiated bulk polymerization when light transmission may be restricted, whereas Filtek Z250 is conventionally placed and photoactivated in smaller increments. The two materials therefore differ not only in composition and curing mechanism but also in their intended handling and clinical application. Clinical material selection should consider cavity geometry, curing-light accessibility, increment thickness, mechanical demands, handling characteristics, adhesive compatibility, and the full body of physical and clinical evidence.

A strength of the present study was the standardized comparison of two materials with different polymerization mechanisms using complementary chemical and mechanical outcomes. We standardized specimen dimensions, curing conditions, measurement surfaces, storage conditions, and testing procedures, and used multiple time points to characterize DC development rather than relying solely on immediate measurements.

Several limitations should be acknowledged. First, we evaluated only one chemical cure and one light-cure composite; therefore, the findings cannot be generalized to all materials within either curing category. Second, the materials differed substantially in resin chemistry and filler loading, preventing us from fully separating the effects of the curing mechanism from those of composition. Third, ATR-FTIR measurements were limited to the bottom surface of 2-mm specimens, and no top-surface or depth-dependent measurements were performed. Fourth, the DC observation period was limited to 24 h, and long-term polymer-network maturation was not assessed. Fifth, the study evaluated only DC; it did not assess other clinically relevant properties, including microhardness, flexural strength, fracture toughness, wear resistance, polymerization shrinkage, water sorption, solubility, and interfacial behavior. Finally, the in vitro design cannot reproduce the combined thermal, mechanical, chemical, and biological conditions encountered clinically.

Future investigations should include several chemical cure, dual-cure, and light cure composites. They should use materials with more closely matched filler contents to better isolate the influence of the polymerization mechanism and evaluate DC at the top and bottom surfaces of clinically relevant increments, with longer observation periods.

Additional assessment of microhardness, flexural properties, fracture resistance, wear, water sorption, solubility, monomer elution, polymerization shrinkage, and bond performance would provide a more comprehensive characterization of chemical cure bulk-fill composites. Ultimately, more clinical studies are required to determine whether their depth-independent initiation translates into satisfactory long-term restorative performance.

## 5. Conclusions

Under the conditions of this in vitro investigation, Filtek Z250 achieved higher DC values throughout the common assessment periods. Although conversion continued to increase in both materials up to 24 h, the chemical cure composite remained below the values recorded for the light-cured material. The final behavior of resin-based composites is also likely influenced by their formulation, inorganic content, and the characteristics of the resulting polymer network.

These findings apply only to the materials, specimen dimensions, measurement surface, and testing conditions used in the study. A broader evaluation of chemical cure bulk-fill composites should include additional products, measurements at varying depths, longer follow-up periods, using different aging procedures, and measurement of additional physico-mechanical properties before establishing clinical recommendations.

## Figures and Tables

**Table 1:** Investigated materials.

Material	Manufacturer	Classification	Organic Matrix	Filler Composition	Filler Loading
Stela	SDI, limited, Bayswater, Victoria, Australia	Chemical cure bulk-fill composite	Urethane Dimethacrylate (UDMA) Glycerol Dimethacrylate (GRMA)	Fumed silica, barium alminoborsilicate glass, fluoro aminosilicate glass, ytterbium triflourde, calcium aluminate	61.2 wt% (36.4 vol%)
Filtek™ Z250	3M ESPE, St. Paul, MN, USA	Nano-Hybrid Light-cured composite	Bisphenol A Glycidyl Dimethacrylate (Bis-GMA) Urethane Dimethacrylate (UDMA) Bisphenol A Ethoxylated Dimethacrylate (Bis-EMA)	Zirconia/silica filler system particles size range 0.01–3.5 μm	82 wt% (60 vol%)

**Table 2:** Between-material comparison of DC at shared post-initiation assessment times. T0 represents the material-specific unpolymerized reference condition used for DC calculation and was not considered a post-initiation outcome. The material-specific immediate measurements were excluded from the direct between-material comparison because they represent different stages of polymerization. Therefore, between-material statistical comparisons were restricted to the four shared post-initiation assessment (5 min, 10 min, 20 min, 24 h).

I Time	Filtek Z250	Stela	Mean Difference	Approximate *p*-Value
5 min	57.35 ± 4.56%	40.57 ± 2.22%	16.78 (14.93–18.64)	<0.001
10 min	59.06 ± 4.49%	44.79 ± 3.08%	14.27 (12.28–16.26)	<0.001
20 min	59.34 ± 4.57%	45.37 ± 3.06%	13.97 (11.96–15.98)	<0.001
24 h	62.39 ± 4.23%	47.58 ± 3.04%	14.81 (12.90–16.71)	<0.001

## Data Availability

The original contributions presented in the study are included in the article, further inquiries can be directed to the corresponding author.
